# Industry Perspective on Current Guidelines, Challenges, and Future Directions for ADME Characterization of Payloads for Antibody‐Drug Conjugates

**DOI:** 10.1002/prp2.70349

**Published:** 2026-11-06

**Authors:** Vijay More, Kiran Deshpande, Chaitali Passey, Gaurav Bajaj, Jeff Barrett, Manish Gupta

**Affiliations:** ^1^ Translational and Quantitative Sciences Genmab Inc. Plainsboro New Jersey USA

## Abstract

A total of 15 antibody‐drug conjugates (ADC)s have been approved by USFDA till December 2025 with the use of nine cytotoxic payload molecules. Small molecule payloads can undergo enzymatic or nonenzymatic biotransformation, distribution, protein or red blood cell binding, transporter mediated disposition and urinary and/or biliary‐fecal excretion. Existing regulatory framework suggests payloads to be characterized for absorption, distribution, metabolism and elimination (ADME) like small molecule therapeutics. Additionally, drug–drug interactions (DDI) relevant investigations are imperative for the payloads. This review focuses on different approaches sponsors have adopted to address payload specific ADME‐DDI questions. The comparison of ADME characteristics of nine approved payloads is presented. It is pointed out that because of lack of ADC specific guidance from regulators, as well as novelty of the ADC platforms, there is no uniformity in addressing ADME properties of payloads, and noticeable differences in approaching the necessity of providing clinical drug interaction data. As ADCs evolve, and more payloads are being developed, the regulatory framework will also need to evolve to guide developers.

## 1 Introduction

Antibody drug conjugates (ADC) deliver potent cytotoxic small molecule payloads to tumor cells through targeted antibodies. This helps in reducing off‐target adverse effects usually accompanied by the administration of chemotherapy. ADCs are comprised of targeted immunoglobulin and a linker molecule that facilitates conjugation of payload. The number of payloads that can be conjugated to an antibody depends on the linker technology, and it is referred to as drug‐antibody ratio (DAR). Cleavable linkers are designed to release the payload molecule upon specific enzymatic catabolism or specific conditions, such as pH changes, whereas noncleavable linkers release payload upon complete breakdown of the ADC in a nonspecific manner [1]. Till December 2025, USFDA has approved 15 ADC therapies in oncology. Table 1 provides details of all USFDA approved ADCs till December 2025, including payload, mechanism of action, DAR, and linker information. This review specifically addresses USFDA approved ADCs, and not the products that have been approved by other regulators like PMDA or CDE but are not yet approved in the United States. The ADCs that are not considered in this review are trastuzumab rezetecan, sacituzumab tirumotecan, trastuzumab botidotin, becotatug vedotin, izalontamab brengitecan (China only approvals), and cetuximab sarotalocan (Japan only approval).

**TABLE 1:** List of approved ADC compounds (until December 2025).

Compound	Trade name	Target	Payload	Linker	Payload action	DAR	Approval
Brentuximab vedotin [2, 3]	Adcetris	CD30	MMAE	Protease cleavable, maleimidocaproylvaline‐citrulline‐p‐aminobenzyloxycarbonyl (MC‐VCPABC)	Microtubule inhibition	4.0	USFDA 2011
Polatuzumab vedotin [4]	Polivy	CD79b	MMAE	Same as brentuximab vedotin	Microtubule inhibition	3.5	USFDA 2019
Enfortumab vedotin [5, 6]	Padcev	Nectin‐4	MMAE	Same as brentuximab vedotin	Microtubule inhibition	3.8	USFDA 2019
Tisotumab vedotin‐tftv [2, 7]	Tivdak	Tissue factor	MMAE	Same as brentuximab vedotin	Microtubule inhibition	4.0	USFDA 2021
Telisotuzumab vedotin‐tllv [8]	Emrelis	cMET	MMAE	Same as brentuximab vedotin	Microtubule inhibition	3.1	USFDA 2025
Belantamab mafodotin‐blmf [2, 9]	Blenrep	BCMA	MMAF	Maleimidocaproyl Noncleavable	Microtubule inhibition	3.7–4.0	USFDA, 2020, withdrawn 2022, approved again 2025
Trastuzumab emtansine [2, 10]	Kadcyla	HER2	DM1	Succinimidyl trans‐4‐(maleimidylmethyl) cyclohexane‐1‐carboxylate	Microtubule inhibition	3.5	USFDA 2013
Mirvetuximab soravtansine [2, 11, 12]	Elahere	FRα	DM4	Sulfo‐SPDB (1‐(2,5‐dioxopyrrolidin‐1‐yl)oxy1‐oxo‐4‐(pyridin‐2‐yldisulfanyl)butane‐2‐sulfonic acid) Enzyme cleavable	Microtubule inhibition	3.5	USFDA 2022
Trastuzumab deruxtecan [2, 13]	Enhertu	HER2	Dxd	mc‐Gly‐Gly‐Phe‐Gly Enzyme cleavable	Top1 inhibition	7.8	USFDA 2019
Datopotamab deruxtecan [14]	Datroway	Trop‐2	Dxd	Same as trastuzumab deruxtecan	Top1 inhibition	4.0	USFDA 2025
Sacituzumab govitecan [2, 15]	Trodelvy	Trop‐2	SN38	CL2A linker Acid cleavable	Top1 inhibition	7.6	USFDA 2020
Loncastuximab tesirine‐lpyl [2, 16]	Zynlonta	CD19	SG3199	PEG‐Val‐Ala‐ PABC Cleavable	DNA damage	2.3	USFDA 2021
Moxetumomab pasudotox [2, 17]	Lumoxiti	CD22	PE38	Cleavable	Immunotoxin	NA	USFDA 2018
Inotuzumab ozogamicin [2, 18]	Besponsa	CD22	Ozogamicin	AcButDMH Acid cleavable	DNA damage	6	USFDA 2017
Gemtuzumab ozogamicin [2, 19, 20]	Mylotarg	CD33	Ozogamicin	AcButDMH Acid cleavable	DNA damage	2.5	USFDA 2000, withdrawn 2010, approved again 2017

Development of ADCs involves detailed nonclinical and clinical characterization of the antibody, as well as the payload. If the ADC uses a novel linker, then the metabolic characterization of the linker may also be essential. The details of the linker design and its impact on ADC PK are reviewed elsewhere [21]. After internalization of the ADC, the payload is released inside cells and engages in the cytotoxic pathway. Once free inside the cell, the payload is handled similar to small molecule xenobiotics. This involves its release from the cell through passive diffusion or active transport, entry into neighboring tumor or nontumor cells, appearance into systemic circulation, binding to plasma proteins and blood cells, uptake into tissues throughout the body, hepatic biotransformation, renal and/or biliary excretion and/or accumulation in tissues as intact payload and/or a metabolite (Figure 1).

**FIGURE 1:**
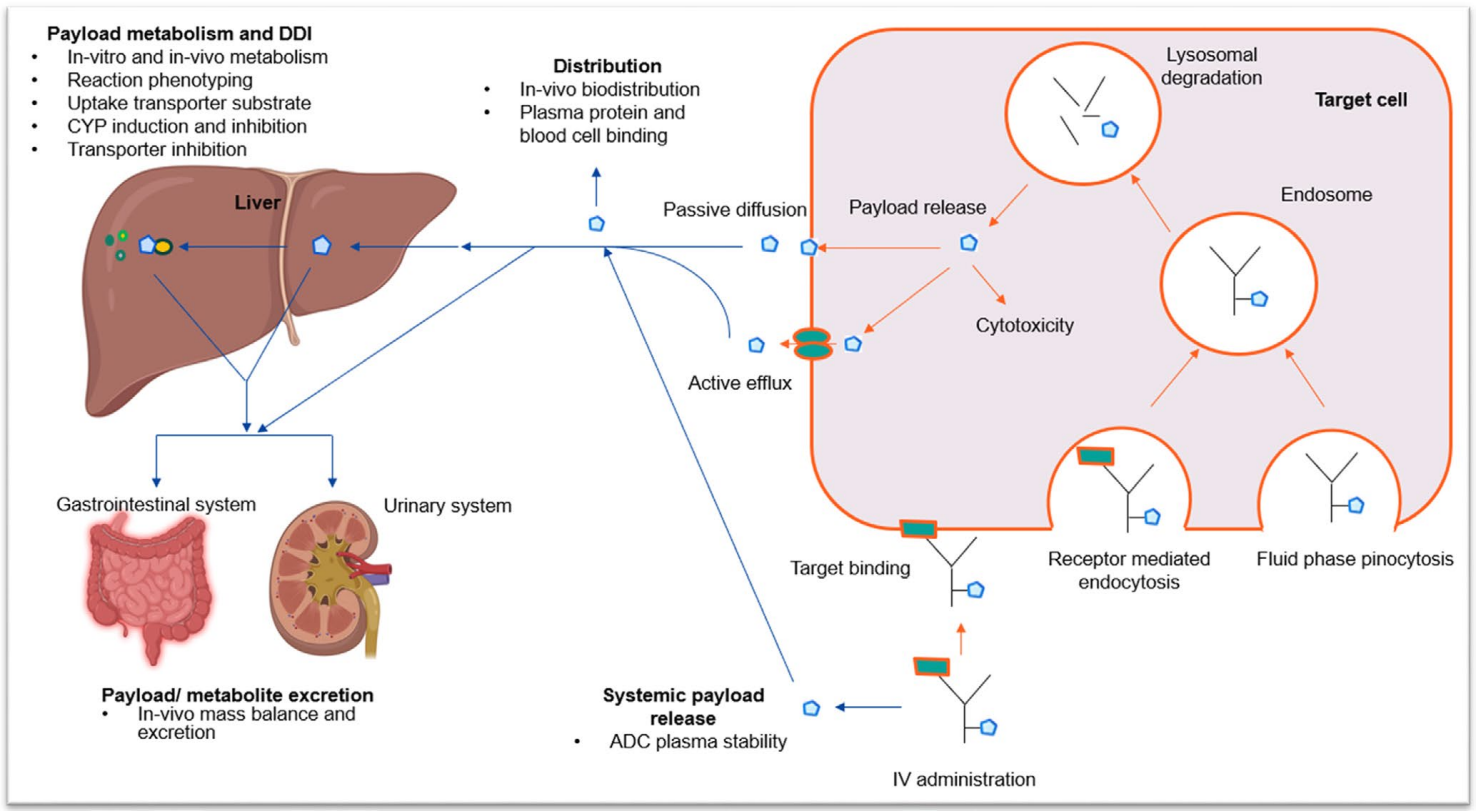
Payload ADME considerations during ADC development.

Given the high cytotoxic potential of the payload, its detailed characterization regarding metabolism and disposition is essential while developing ADC molecules with novel payload. In March 2024, USFDA released its first ever final guidance for clinical pharmacology considerations for ADC development [22]. This guidance provided nonbinding recommendations to the sponsors for ADC development, specifically for cytotoxic payloads. However, for the next generation of noncytotoxic payloads (including immunomodulators and protein degraders), the guidance does not mention any specific recommendations.

This review focuses on the metabolism and drug‐interaction related characterization of the ADC payloads, current regulatory framework, and compilation of the relevant data for marketed payloads including strategies taken by sponsors to develop existing ADCs. Finally, we provide our perspective on the ADME assays for future ADC development.

## 2 Current ADC Regulations

As of December 2025, the development of ADCs globally is guided by USFDA March 2024 and China's Center for Drug Evaluation (CDE)'s “Technical guidance for ADC non‐clinical development” [23] along with “Technical guidelines for clinical pharmacology studies of ADCs” [24] are three dedicated guidelines from regulators. European Medical Agency (EMA), International Council for Harmonization (ICH), Japan Pharmaceuticals and Medical Devices Agency (PMDA) haven't dedicated a guideline yet for ADCs. ICH‐M12 guidance makes a brief mention of ADC's and suggests references to the small molecule DDI strategies for payloads. Even prior to March 2024 guidance release, sponsors utilized the approach of a combination of small molecule relevant nonclinical activities as well as routine biologics development activities to support ADC development.

The antibody backbone of an ADC is expected to be catabolized into peptides and amino acids, thus, not posing any risk for drug‐interactions, QTc prolongation, and metabolite relevant effects. However, inclusion of these studies is imperative for a stand‐alone free small molecule payload which is released from ADC into the circulation. In most instances, guidance suggests the sponsor to refer to small molecule specific guidance for characterizing payload. Detailed ADME (absorption, distribution, metabolism, and elimination) and nonclinical toxicology characterization of payload is expected to be included in the filings for ADCs. However, there are certain challenges with respect to following small molecule guidance during development of ADCs.

In 2014, ADC ADME working group was initiated by the International Consortium for Innovation and Quality in Pharmaceutical Development. The white paper published by this group provided perspective on the ADME studies essential in ADC development. This serves as a preliminary framework which has served as a reference for sponsors to plan ADC development [25].

### 2.1 Metabolism and Mass Balance

For small molecule therapies, metabolism‐related assessments have been standardized based on many years of guidance from regulators. Detailed in vitro metabolite identification in hepatocytes and liver microsomes is performed to specifically understand the contribution of cytochrome P450 (CYP) and non‐CYP mediated metabolism. Once metabolic pathways are identified, reaction phenotyping studies reveal specific involvement of CYP enzymes. In vitro metabolism studies are generally performed in biomaterials from pre‐clinical species as well as human.

In vivo metabolism of small molecules is typically studied in a rodent and a nonrodent preclinical species. This is guided by the toxicologist's choice of species for the GLP safety studies [26]. In vivo metabolism studies are essential to clarify if there are any metabolites that cannot be identified in vitro.

For ADC development, these studies are essential to understand the payload's potential to be metabolized in the body after ADC administration, as well as the relevance of the nonclinical safety studies. FDA guidance uses vague language to mention the necessity of payload metabolism data, as well as enzyme information, and guides sponsors to follow small molecule guidance whenever possible.

For small molecule therapies, human mass balance and metabolism studies are conducted to specifically generate data for human metabolism. This is usually a requirement for marketing authorization and is performed prior to New Drug Application (NDA) [27]. In the human radiolabeled mass balance study guidance from September 2024, USFDA clarified that it's not feasible for ADCs, given the possibility of distributing cytotoxic payload through the subject's body. This is a major point of distinction for regulations of small molecule development versus ADC development. A review by Rudolph et al. [28] suggested advanced microtracer approaches to counter the cytotoxic payload distribution in patients with conventional mass balance studies. The approach is described along with a few examples of small molecule programs and biologics that use microtracer study design. It should be noted that none of the approved ADCs till the end of 2025 have used this approach for clinical mass balance studies.

In absence of definitive clinical mass balance and metabolism data for ADCs creates a need for more thorough in vitro human biomaterial experimentation. Clinical mass balance guidance does mention the need of detailed in vitro payload metabolism for ADCs [27], but the actual ADC specific guidance does not delve into any details of these suggested experiments [22]. Section 3 of this review summarizes the in vitro studies performed by the sponsors to address this issue. Analysis of metabolites from human plasma and excreta can provide useful information in lieu of clinical mass balance studies.

### 2.2 Distribution

For small molecules therapeutics, preclinical quantitative whole‐body autoradiography (QWBA) experiments are conducted to assess drug distribution and accumulation in tissues. These studies are usually conducted in rodent species. For ADCs, distribution is a multi‐faceted issue where the design and chemical make‐up of the linker, target expression, and payload‐specific physicochemical properties can influence the distribution [29, 30, 31, 32].

According to the S9 nonclinical evaluation of anticancer pharmaceuticals guidance (question and answer section), distribution studies are not warranted for the ADCs [33]. One of the important goals of preclinical QWBA studies is to perform dosimetry calculations for informing clinical mass balance studies. However, since clinical mass balance studies are not usually performed for ADCs, it's understandable that preclinical QWBA studies are not recommended. Review of publications suggests that sponsors perform QWBA or other types of distribution studies for payloads or ADC conjugated with radioactive payload in preclinical species. For novel linker systems or novel payloads, understanding tissue accumulation is critical, and suggestion from regulators will be helpful.

### 2.3 Drug Interactions

USFDA, amongst other regulators, have clearly defined DDI guidance for small molecule development. In January 2020, USFDA released two separate documents outlining in vitro drug interactions and clinical drug‐interactions for small molecules. In August 2024, USFDA adapted ICH M12 drug‐interactions guidance as a final version [34]. These guidelines are specific, detailed, and address the CYP enzymes as well as transporter studies comprehensively. M12 guidance defined thresholds for enzyme induction, inhibition, transporter inhibition, along with providing useful information on experimental systems, appropriate controls and validations [34].

USFDA also released a DDI guidance for therapeutic proteins in June 2023 [35], covering the protein mechanism relevant to inflammatory cytokines and noninflammatory mechanisms. ADC drugs pose the risk for DDI from both antibody as well as payload portion. The M12 guidance specifically mentions evaluation of payload similar to small molecules and antibody's potential for DDI [34]. Clinical investigation of DDI for small molecules is motivated by in vitro data thresholds. DDI risk for ADC payload as a precipitant is minimized due to low circulating plasma levels. However, DDI potential of payload as a substrate needs to be clearly defined. M12 guidance urges sponsors to understand exposure‐response relationship for all components of ADC prior to making decisions about clinical DDI studies. Brief mention of this criterion is not as conspicuous as in case of small molecule drugs. Inferring from the M12 guidance, for an ADC, if exposure‐response (efficacy and/or safety) relationship for free payload is relevant, then DDI studies of payload as a substrate may be needed. International Consortium for Innovation and Quality in Pharmaceutical Development published a perspective on risk‐based DDI evaluations for ADCs [36]. According to the summary published in this white paper, the substrate risk is considered greater compared to precipitant risk for payloads, which aligns with the language in the ICH M12.

Of the 15 approved ADCs, which featured a total of nine novel payloads, clinical DDI studies were conducted only for two ADCs, brentuximab vedotin and trastuzumab deruxtecan [37, 38].

### 2.4 Organ Impairment

Intrinsic factors such as hepatic or renal impairment in patients can significantly alter PK of payload and ADC, leading to safety or efficacy issues. USFDA recommends careful consideration of these special populations during clinical development of an ADC [22]. Based on in vitro and preclinical experimentation, sponsors have clear idea of payload and/or metabolite elimination pathways after administration of an ADC. This knowledge assists in planning dedicated organ impairment trials or population PK analysis of clinical data obtained from safety/efficacy directed trials.

For hepatic impaired population, USFDA makes a reference to a dedicated 2003 guidance [39] that provides detailed recommendations for clinical study design, data analysis and interpretations. Results from these studies guide sponsors in dose modification for special populations, or labeling criteria that advise careful administration or exclusion of patients from ADC administration.

For organ impaired populations, rather than adding exclusion criteria based on in vitro/preclinical data, USFDA recommends specific studies and data analysis. Sponsors are suggested to refer to July 2020 guidance on “diversity in clinical trials” document [40]. USFDA also released draft guidance for hepatic impaired patient PK which provided some modifications to the study design and hepatic function measurement criteria.

## 3 ADME Data Review for Approved ADCS

### 3.1 Payload In Vitro ADME Characterization

Tabulated summaries of data from in vitro ADME assays for payloads linked to approved ADCs are presented in Table 2 for plasma protein binding and blood to plasma partitioning ratio, Table 3 for metabolizing enzymes, and Table 4 for drug transporters.

**TABLE 2:** Protein binding and blood plasma partitioning of ADC payloads reported in BLA multi‐disciplinary review.

Payload	Plasma protein binding % (assay concentration)	Blood: plasma partitioning
MMAE	67.9 (1 nM), 77.5 (10 nM), 82.2 (100 nM)	0.79–0.98
MMAF	55.5 (0.5 ng/mL), 69.7 (5 ng/mL), 84.8 (50 ng/mL)	Not reported
DM1	93.2 (20 ng/mL), 91.8 (100 ng/mL), 92.5 (1000 ng/mL)	Not reported
DM4	99.4 (not reported)	0.907–1.62
Dxd	98 (10 ng/mL), 97.4 (30 ng/mL), 96.8 (100 ng/mL)	0.6
SN38	94–96 (50 to 200 ng/mL)	1.23–1.46
SG3199	95 (0.8‐50 ng/mL)	Not reported
Ozogamicin	97 (not reported)	Not reported

**TABLE 3:** Interactions of approved ADC payloads with metabolizing enzymes reported in BLA multi‐disciplinary review.

Payload	Metabolizing enzyme	Enzyme induction	Enzyme inhibition
MMAE	CYP3A4 major and CYP2D6 minor	Not an inducer of CYP enzymes	Direct inhibition of CYP3A4/5 (IC_50_ = 10 μM) Time‐dependent inhibition of CYP3A4/5 (Kinact = 0.1/min) Little or no direct inhibition of CYP1A2, 2B6, 2C8, 2C9, 2C19, and 2D6
MMAF	Major metabolism is nonenzymatic, with minor pathways of oxidation and conjugation. No specific enzymes listed for minor pathways	Not an inducer of CYP enzymes	Not an inhibitor of CYP enzymes
DM1	CYP3A4/5 major, CYP2C9 and CYP2E1 minor [41]	Not an inducer of CYP enzymes	No direct inhibition of any CYP enzymes until 678 nM Time‐dependent inhibition of CYP3A4 at IC_50_ = 155 nM
DM4 (and its active metabolite S‐methyl DM4)	Both DM4 and S‐methyl DM4 are substrates of CYP3A4	DM4 and S‐methyl‐DM4 are not inducers of CYP enzymes	DM4 and S‐methyl‐DM4 showed no direct inhibition on CYP1A2, CYP2B6, CYP2C8, CYP2C9, CYP2C19, CYP2D6, or CYP3A4/5 DM4 showed time‐dependent inhibition of CYP3A4, and S‐methyl DM4 showed weak time‐dependent inhibition of CYP2C8 and CYP2C9.
Dxd	CYP3A4 [42]	Not an inducer of CYP enzymes	Not an inhibitor of CYP enzymes
SN38	UGT1A1	Not reported	CYP3A4 direct inhibition (*K*_i_ = 121 μM) CYP3A4 time dependent inhibition (*K*_inact_ = 0.1/min) CYP2A6 inhibition (*K*_i_ = 181 μM) CYP2C9 inhibition (*K*_i_ = 156 μM) [43]
SG3199	CYP3A4/5 is potentially involved (no experimental data reported)	Not reported	Not an inhibitor of CYP enzymes
Ozogamicin	Nonenzymatic reduction	Low potential to induce the activities of CYP1A2, CYP2B6, and CYP3A4 at clinically relevant concentrations	Low potential to inhibit the activities of CYP1A2, CYP2A6 (tested only using gemtuzumab ozogamicin), CYP2B6, CYP2C8, CYP2C9, CYP2C19, CYP2D6, and CYP3A4/5 at clinically relevant concentrations Low potential to inhibit the activities of UGT1A1, UGT1A4, UGT1A6, UGT1A9, and UGT2B7 at clinically relevant concentrations

**TABLE 4:** Interactions of approved ADC payloads with drug transporters reported in BLA multi‐disciplinary review.

Payload	Substrate	Inhibitor
MMAE	Substrate of P‐gp Not a substrate of BCRP, MRP2, OATP1B1, OATP1B3	Not an inhibitor of P‐gp, BSEP, BCRP, MRP2, OCT1, OCT2, OAT1, OAT3, OATP1B1, OATP1B3
MMAF	Substrate of OATP1B1, OATP1B3, MRP1, MRP2, MRP3, BSEP, P‐gP	Not reported
DM1	Substrate of MDR1	Not reported
DM4	DM4 and S‐methyl DM4 are substrate of P‐gp	DM4 and S‐methyl‐DM4 do not inhibit P‐gp
Dxd	Substrate of OATP1B1, OATP1B3, MATE‐2 K, MRP1, BCRP and P‐gp	Inhibits OAT1 (IC_50_ = 12.7 μmol/L) and OATP1B1 (IC_50_ = 14.4 μmol/L). Does not inhibit OAT3, OCT1, OCT2, OATP1B3, MATE1, MATE2K, P‐gp, BCRP, and BSEP.
SN38	Substrate of P‐gP and MRP1 per a publication [44], not reported in the BLA	Not reported
SG3199	Substrate of P‐gp but not BCRP, OATP1B1, OATP1B3 or OCT1	Inhibitor of BCRP, OATP1B1, OAT1, OAT3, OCT1, OCT2, MATE1, MATE2K, BSEP (IC_50_ for all above clinical exposure) Not an inhibitor of P‐gp and OATP1B3
Ozogamicin	Substrate of P‐gp but not BCRP, OATP1B1 and OATP1B3	Not an inhibitor of P‐gp, BCRP, OAT1, OAT3, OCT2 Minimal inhibition of OATP1B1 and OATP1B3 (30% and 16% inhibition at 0.1 mM concentration, respectively)

MMAE shows concentration dependent increase in protein binding, with highest binding observed up to 82.2%. Similarly, MMAF has protein binding as high as 84.8% at highest concentration tested, and as low as 55.5% at the lowest concentration tested. Payloads DM1 and DM4, as well as Dxd, SN‐38, SG3199 and ozogamicin are all highly plasma protein bound in in vitro assays. In contrast, blood to plasma ratios are reported for MMAE, DM4, Dxd and SN38, whereas other payloads do not report this data in their regulatory filings. MMAE and Dxd show priority for plasma, whereas DM4 and SN38 partition more into blood in vitro. MMAE demonstrated higher blood partitioning in preclinical species compared to human (published by Ref. [45], not mentioned in the FDA review files for brentuximab vedotin).

The payload for moxetumomab pasudotox is an exotoxin called PE38 and given that it's not a typical small molecule payload, its in vitro ADME characterization is not reported.

MMAE is primarily metabolized by CYP3A4, similar to DM1, DM4, and Dxd (Table 3). MMAF and ozogamicin are eliminated via nonenzymatic catabolism, and SN38 is the only payload that is primarily metabolized by UGT1A1. Although regulatory filings for SN38 address UGT1A1 involvement in metabolism in detail, the sponsor also mentioned the possibility of interactions with CYP3A inhibitors, although no experimental data for CYP3A involvement was presented in the BLA. For the payload of mirvetuximab sorvatansine, DM4, the ADME assays are reported for DM4 and its active metabolite S‐methyl‐DM4. As DM4 is metabolically unstable, its conversion to S‐methyl‐DM4 is rapid. Additionally, the metabolite has greater cytotoxic potential, making it a significant small molecule catabolite that requires proper ADME characterization.

Of all approved ADC payloads, ozogamicin is the only payload that demonstrated CYP1A2, 2B6, and 3A4 enzyme induction potential at clinically relevant concentrations. MMAE, MMAF, DM1, DM4, S‐methyl‐DM4, and Dxd do not induce any CYPs tested in vitro. CYP induction data is not reported for SG3199, SN38, and PE38.

CYP enzyme inhibition assays are reported for all payloads except PE38. Along with direct inhibition (as IC_50_), time‐dependent inhibition (as K_inact_) has also been reported for all major CYP isoforms in payload ADME characterization. Reporting UGT conjugation enzyme inhibition is not a common practice for small molecule therapeutics. Similarly, the only payload for which UGT inhibition was reported is ozogamicin. MMAE and SN38 are direct and time‐dependent inhibitors of CYP3A4/5, whereas DM4 inhibited CYP3A4 only in time‐dependent assay.

For drug transporter interaction of the payloads, the regulatory documents have consistently provided a summary of the substrate data. All the approved payloads are substrates of the P‐gp efflux pump except PE38. Additionally, MMAF is a substrate of OATP1B1, OATP1B3, MRP1, MRP2, MRP3, and BSEP. For DM1 and DM4, the substrate data are only reported for P‐gp, but not for any other transporters. Payload Dxd is a substrate for OATP1B1, OATP1B3, MATE2K, MRP1, and BCRP, along with being a substrate for P‐gp. SN38 is a substrate of P‐gp and MRP1; however, this information is not reported in the BLA document for Sacituzumab govitecan, but was cited here from a peer‐reviewed publication [43].

For transporter inhibition, the information is not available for all payloads. For ADCs with MMAF, DM1 or SN38, the transporter inhibition data are not reported in the BLA. MMAE is not an inhibitor of any transporters tested. Dxd minimally inhibits OAT1 and OATP1B1; however, the IC_50_ values reported are far higher compared to the reported clinical concentration of the payload after trastuzumab deruxtecan administration. SG3199 also inhibits BCRP, OATP1B1, OAT1, OAT3, OCT1, OCT2, MATE1, MATE2K, and BSEP but not at clinically relevant concentrations after administration of locanstuximab tesirine. Transporter interactions for PE38 are not reported as it's not a typical small molecule.

### 3.2 Payload Preclinical In Vivo ADME Characterization

Given the possibility of having a disconnect between in vitro and in vivo studies, alignment of metabolic pathways with in vivo data for a new chemical entity is expected.

Brentuximab vedotin was the first USFDA approved ADC with MMAE as a payload. Mass balance and excretion study of radioactive MMAE was performed in Sprague Dawley rats after intravenous bolus dose. This was followed by human excretion study with nonlabeled ADC. Additionally, the metabolite identification was also reported for excretory matrix in clinical study. MMAE is primarily eliminated by fecal route in preclinical species. Performing mass balance and metabolite identification in rat and/or monkeys is routinely reported for the ADC regulatory filings. For all other MMAE based ADCs subsequent to brentuximab vedotin, mass balance studies were not performed separately. Instead, data from approved brentuximab vedotin were referenced (Table 5).

**TABLE 5:** Summary of in vivo ADME study designs reported in the BLA multi‐disciplinary review for ADCs approved by USFDA.

ADC	In vivo metabolism study	In vivo distribution study
Brentuximab vedotin	Rat: mass balance, metabolism and excretion with radioactive unconjugated payload and ADC conjugated with radioactive payload Monkey: no in vivo study Human: Urine and fecal metabolite identification using nonradioactive ADC in target patient population Fecal excretion as primary elimination, and intact MMAE was preliminary excretion moiety. Across species, 13 metabolites were identified	Rat: distribution of unconjugated payload (detailed design and data not mentioned in BLA). MMAE related radioactivity distributed to multiple tissues and cleared rapidly except in uveal track
Polatuzumab vedotin	Not performed in any species	No in vivo distribution study reported
Enfortumab vedotin	Rat: mass balance and excretion of radioactive unconjugated payload. MMAE was mostly excreted via fecal route and as an unchanged moiety	Rat: radioactive unconjugated payload distribution study. MMAE related radioactivity was well distributed and mostly cleared in 96 h
Tisotumab vedotin	Not performed in any species	Monkey: biodistribution study of ADC was conducted using PET imaging. Highest distribution in liver, bone marrow, lungs, kidneys, heart, small intestine, and gall bladder. No gender difference in distribution
Belantamab mafodotin	Rat: mass balance, metabolism and excretion of radioactive unconjugated payload. About 83% fecal and 13% urine excretion of payload related radioactivity. Mostly unchanged payload excreted. < 5% metabolism.	Rat: biodistribution of ADC using fluorescent label on antibody and no label on payload. Biodistribution of antibody was not affected by presence or absence of payload. Antibody showed distribution in eye, eyelids, lacrimal and harderian glands, and liver
Trastuzumab emtansine	Rat: mass balance, metabolism and excretion study following administration of ADC with radioactive label on payload. Fecal excretion predominant, most DM1 was eliminated as disulfide bound conjugate. About 1% of administered dose was excreted as unchanged DM1.	Rat: tissue distribution study using radiolabeled antibody conjugated with nonradioactive payload. Radioactivity was highest in blood followed by lungs, liver, kidney, heart, bone marrow, and spleen. No distribution to brain
Mirvetuximab soravtansine	Preclinical excretion and metabolism studies in rats. Primary excretion in feces as several metabolites of lower abundance Human plasma quantification was mentioned for unconjugated payload (DM4) and a major metabolite (S‐methyl DM4)	Distribution study of tritiated payload conjugated to cold antibody. Liver, bone marrow, lung, kidney, and spleen with higher distribution. Eyes show slower clearance compared to plasma. Some drug penetration in testes and brain
Trastuzumab deruxtecan	Monkey: metabolism and excretion of ADC after administration of radioactive payload conjugated to nonradioactive antibody. About 67.3% fecal and 18.7% urinary excretion. Unchanged payload was the only detectable moiety in excreta	Monkey: tissue distribution study using radiolabeled payload conjugated to radioactive antibody. Besides blood and intestinal cavity, major radioactivity was distributed to kidney, lungs, and liver. No retention in any tissue
Datopotamab deruxtecan	Rat and monkey: mass balance, metabolism and excretion of radioactive payload. No studies with ADC. Primary fecal excretion, and as unchanged payload in rats. Similar excretion in monkeys, with minor identification of Dxd metabolites (~5%)	Rat and monkey: tissue distribution study using radiolabeled payload. No studies with ADC. Radioactivity quickly distributed and cleared without any retention in rats and monkeys
Sacituzumab govitecan	No in vivo metabolism or excretion study reported	No in vivo distribution studies reported
Gemtuzumab ozogamicin	No in vivo mass balance or metabolism studies reported	No in vivo distribution studies reported
Inotuzumab ozogamicin	Preclinical excretion studies are reported without specifying species or study design details Detection of a major metabolite of payload is reported in patients	Rat: distribution study is described without specifying use of radio or another kind of label
Loncastuximab tesirine	Rat: mass balance, metabolism and excretion study was reported after administration of radioactive unconjugated payload. Feces was the major route of excretion	Rat: tissue distribution was studied in rats after administration of radioactive unconjugated payload. Rapid distribution to majority of tissues, no detection in melanin containing tissues, brain or spinal cord
Moxetumomab pasudotox	No in vivo metabolism studies are reported	No in vivo distribution studies are reported

MMAF based ADC, belantamab mafodotin reported mass balance study in rats after administering radioactive payload. For distribution, fluorescent labeled ADC or naked antibody were administered to rats. Distribution study also included an arm with tissue specific quantification of free payload. There were no reported monkey studies for mass balance or distribution for belantamab mafodotin. MMAF is primarily eliminated through fecal excretion similar to MMAE.

For trastuzumab emtansine, rat mass balance study was performed with free radioactive payload. Use of both bile‐duct canulated (BDC) as well as intact rats was reported. In a separate study, administration of antibody conjugated with radioactive DM1 was reported as well in male and female rats. The distribution studies were also reported in normal rats using radioactive antibody conjugated with cold payload. This interesting distribution study design yielded data on the biodistribution of antibody in the rats, but distribution of payload was not addressed. In fact, the comparator groups were radioactive antibody alone vs. radioactive antibody conjugated to payload. So, the influence of payload on antibody distribution was addressed. As referenced from the regulatory documents, trastuzumab emtansine did not report any mass balance or distribution studies in monkeys.

In case of mirvetuximab sorvatansine, antibody conjugated with radioactive payload was used for mass balance study in intact and BDC rats. In monkeys, free cold payload plasma PK study was reported in the excretion section. Proper radioactive mass balance and metabolism was not reported in monkeys. For distribution of mirvetuximab sorvatansine, antibody conjugated with radioactive payload was used in rats. Distribution studies were not reported in monkeys.

For trastuzumab deruxtecan, metabolism and distribution studies reported are more comprehensive. Radioactive free payload mass balance and metabolism was reported in intact and BDC rats. Antibody conjugated to radioactive payload was used in monkey mass balance and metabolism study. The distribution study for trastuzumab deruxtecan utilized radiolabeled antibody conjugated to cold payload and compared to antibody conjugated to radioactive payload administered to monkeys.

There were no standalone mass balance and excretion studies reported for sacituzumab govitecan in vivo. The distribution study was a part of PKPD study in xenograft mice bearing squamous cell lung carcinoma, and utilized label on the antibody portion of the ADC. In a separate study in a different tumor bearing mouse model, cold ADC was injected to measure free and conjugated payload concentrations in tissues like liver, small intestines and tumor. In addition to payload, this study also tracked glucuronide metabolite of the payload during the distribution assessment.

Gemtuzumab ozogamicin reported animal studies for elimination stating hepatobiliary excretion of the payload. Details of species, dose levels and tracer labeling were not specified. Distribution study was not reported for gemtuzumab ozogamicin, however for inotuzumab ozogamicin, a rat distribution study was reported. Moreover, the metabolite information was also reported in patients treated with inotuzumab ozogamicin. For both ozogamicin based ADCs, human mass balance studies were not conducted.

Loncastuximab tesirine reported rat mass balance and excretion study using radioactive free payload. Preclinical distribution studies were not reported.

As expected, moxetumomab pasudotox did not report any in vivo metabolism or excretion or distribution studies, given its payload is a peptide, and it expected to catabolize similar to an antibody.

### 3.3 Clinical Drug Interaction Studies for ADCs

Of all the approved ADCs, only two have reported clinical drug interactions studies that were performed during the clinical investigations; brentuximab vedotin and trastuzumab deruxtecan. For other ADCs that use the same payloads, the prescribing information referred to the clinical investigations from earlier products. For several other ADCs with distinctly new payloads, the sponsors used a variety of approaches to address drug interactions in the absence of clinical DDI data.

Based on in vitro studies, MMAE is a substrate, as well as an inhibitor of CYP3A enzyme. Brentuximab vedotin DDI trial assessed 56 patients dosed at 1.2 or 1.8 mg/kg intravenously for a 21‐day cycle. Each patient also received a sensitive CYP3A substrate (midazolam), an inducer (rifampin), or a strong inhibitor (ketoconazole). PK data from patients was assessed to conclude that brentuximab and MMAE are neither inducers nor inhibitors of CYP3A at clinically relevant concentrations. However, MMAE showed interactions as a victim of CYP3A4 inhibition and induction. Similarly, MMAE PK was also impacted by Pgp inhibition in patients. This resulted in labeling recommendations to avoid concomitant use of strong CYP3A4 inhibitors or inducers and P‐gp inhibitors [46, 47].

For clinical DDI of trastuzumab deruxtecan, the study was a Phase I study where patients were dosed at 5.4 mg/kg every 3 weeks. According to in vitro data, payload Dxd is a substrate of CYP3A, OATP1B1, and Pgp. The patients were assessed in the Phase 1 clinical trial for DDI of OATP1B1/CYP3A. As a part of this study, 26 patients were assessed for impact on PK with compounds ritonavir or itraconazole in HER‐2 expressing tumors. Both drugs, in combination with trastuzumab deruxtecan, did not show any significant impact on the PK of payload or ADC. This was reflected in the prescribing information being free of any mention for avoidance of the CYP3A, OATP1B, or Pgp inhibitor concomitant medications [48, 49].

Trastuzumab emtansine and mirvetuximab sorvatansine launched two novel maytansinoid derivate payloads, DM1 and DM4, respectively. Both DM1 and DM4 are substrates for CYP3A according to the published literature, and DM4 also has demonstrated time‐dependent inhibition of CYP3A4. Despite these possible drug interactions, clinical DDI studies were not reported for either of the ADCs. An independently published study reported clinical DDI for trastuzumab emtansine, and it describes the interaction between tacrolimus and DM1. The study showed an increase in the concentration of tacrolimus due to interaction with T‐DM1 [50].

### 3.4 Physiological Based Pharmacokinetic (PBPK) Modeling and DDI Prediction

PBPK models are based on principles of mass balance and focus on developing a deep understanding of the accumulation and disposition of a compound based on pre‐clinical data to provide predictions of drug concentration in plasma and tissue assuming the body is a closed system [51]. These models are powerful tools to assess predicted ADME of novel therapeutics like the ADC specifically for the payloads.

In some situations, the sponsor can choose to run a clinical study with a strong CYP3A4 inhibitor co‐administered with the ADC. This data can be coupled with a PBPK model to simulate interactions with moderate inhibitors or inducers of CYP3A4. This approach can also be applied to organ impairment assessment with PBPK, where one scenario can be run in clinic (like moderate liver failure), whereas other scenarios can be simulated (mild and severe liver failure) [51, 52].

Polatuzumab vedotin used PBPK modeling to ascertain the impact of drug interactions. Given the use of the same linker‐payload, the data from brentuximab vedotin clinical DDI were leveraged to generate a full body PBPK DDI model and validate it for polatuzumab vedotin at clinically relevant doses of 1.8 and 2.3 mg/kg. This stepwise model development also included the in vitro and in vivo parameters generated for polatuzumab vedotin. The final model predicted that co‐administration of CYP3A strong inhibitors could increase MMAE exposure by 45% and decrease it by 63% if co‐administered with a CYP3A inducer like rifampin [4, 37]. The PBPK‐DDI model for polatuzumab vedotin was developed in lieu of a dedicated clinical study and was used to justify the exclusion of concomitant medications.

Another notable example of model informed decision making was telisotuzumab vedotin where a PBPK model was used to assess DDI liability and included in BLA submission. For model development, neonatal receptor binding affinity and half‐life were obtained from in vitro experiments. MMAE PK with DDI information was curated from published clinical trials for vedotin ADCs [53]. The pre‐clinical data specific to telisotuzumab such as physiochemical characterization, drug antibody ratio, human neonatal Fc receptor binding were utilized. This helped develop a preliminary PBPK model which was calibrated and verified using data from the clinical trial. The DDI scenarios were simulated to predict the impact of CY3A4 inhibitor ketoconazole and inducer rifampin. The model predicted an increase in AUC by 43% when co‐administered with a strong CYP3A4 inhibitor and a 70% decrease with a strong CYP3A4 inducer [8].

The latest example is of datopotomab deruxtecan where the sponsor leveraged the information from trastuzumab deruxtecan. A PBPK model was developed to explain the disposition of the ADC dosed at Q3W 6 mg/kg IV administration which is the recommended dose. The deruxtecan model was developed using small molecule information from trastuzumab deruxtecan (5.6 mg/kg Q3W) by leveraging its preclinical data and was qualified using data from both datopotomab and trastuzumab. Both models were validated using clinical data and were then combined to provide a unified PBPK model for datopotomab deruxtecan. Using this model, the sponsor was able to predict a change in the AUC with an increase of 20% and 24% when administered concomitantly with itraconazole and ritonavir, respectively [14].

PBPK modeling is a useful tool to characterize DDI potential for an ADC when the payload is being re‐used, and clinical DDI data from a previous ADC is available. When it comes to novel payloads, PBPK applications are limited unless clinical data is generated for at least some DDI scenarios.

### 3.5 Impact of Organ Impairment on ADC Payload PK

Payload metabolism and excretion occur primarily through the liver and/or kidneys, and USFDA recommends careful consideration of ADC administration to populations with organ impairment. A dedicated clinical study in patients with hepatic and renal impairment was conducted for brentuximab vedotin (Table 6). Moderate and severe hepatic impairment (as defined by Child‐Pugh score) and severe renal impairment significantly affected MMAE PK, and it was indicated on the product label to avoid use in these populations [55]. For tisotumab vedotin and polatuzumab vedotin, organ impairment studies were not conducted, and data from brentuximab vedotin was cited in the BLA. However, for enfortumab vedotin, despite using the same linker‐payload as brentuximab vedotin, separate renal impairment analysis was provided. Interestingly, the results from this analysis concluded that severe renal impairment does not alter MMAE PK significantly, and dose adjustment was not needed for this population [5]. Telisotuzumab vedotin also presented PopPK analysis for justifying no dose adjustment for moderate renal impairment [8].

**TABLE 6:** Summary of hepatic and renal impairment study designs reported in the BLA multi‐disciplinary review for ADCs approved by USFDA.

ADC	Hepatic impairment study	Renal impairment study	PMR
Brentuximab vedotin	Yes, at reduced dose of 1.2 mg/kg Definition: Child‐Pugh (mild: *n* = 1, moderate: *n* = 5, severe: *n* = 1) Observation: ≥ Grade 3 AE was 6/6 (100%) compared with 3/8 (38%) in patients with normal hepatic function	Yes, at reduced dose of 1.2 mg/kg Definition: patients with mild (CL_cr_ > 50–80 mL/min; *n* = 4), moderate (CL_cr_ 3050 mL/min; *n* = 3) and severe (CL_cr_ < 30 mL/min; *n* = 3) Observation: Grade 3 AE 3/3 (100%) compared to 3/8 (38%) in patients with normal renal function	No
Enfortumab vedotin	No	Yes, at 1.25 mg/kg Definition: mild (creatinine clearance; CL_cr_ > 60–90 mL/min; *n* = 135), moderate (CL_cr_ 30–60 mL/min; *n* = 147) and severe (CL_cr_ < 30 mL/min; *n* = 8) renal impairment Observation: No difference in exposure (AUC) of ADC and MMAE in healthy and impaired	No
Tisotumab vedotin	No	No	No
Polatuzumab vedotin	No	No	No
Telisotuzumab vedotin	No	No	No
Belantamab mafodotin [54]	No	No	Yes. Hepatic and renal impairment
Trastuzumab emtansine	No	No	Yes. Hepatic impairment
Mirvetuximab sorvatansine	No	No	Yes. Hepatic impairment
Trastuzumab deruxtecan	Not a dedicated study, but data from one patient with moderate hepatic impairment was shared	No	No. Absence of clinical DDI with hepatic transporters and CYP3A4 was used as a weight of evidence to suggest that moderate hepatic impairment may have smaller effect on payload PK, and dose modification was not suggested, and PMR was not issued
Datopotamab deruxtecan	Not a dedicated study, but PopPK data from 6 moderate and 1 severe hepatic impaired patient presented	Not a dedicated study, but PopPK data from mild and moderate renal impaired patients	No PMR. FDA suggest monitoring adverse events in moderate hepatic impairment and modify dose if needed
Sacituzumab govitecan	No	No	Yes. Hepatic impairment
Loncastuximab tesirine	No	No	Yes. Hepatic impairment
Inotuzumab ozogamicin	Yes, at 0.8–0.5 mg/m^2^ Definition: Moderate (total bilirubin 1.5 to 3 × ULN and any AST), severe (total bilirubin > 3 ULN and any AST any level) Observation: No difference between mild and normal. Insufficient data for moderate and severe	Yes Dose: 0.8–0.5 mg/m2 Q3W Definition: mild renal impairment CL_cr_; based on the Cockcroft‐Gault formula 60–89 mL/min; *n* = 237, moderate renal impairment (CL_cr_ 30–59 mL/min; *n* = 122), or severe renal impairment (CL_cr_ 15–29 mL/min; *n* = 4), normal renal function (CL_cr_ ≥ 90 mL/min; *n* = 402) Observation: No difference between mild, moderate and severe compared with normal. No data for end stage renal	No
Gemtuzumab ozogamicin	Information not available		
Moxetumomab pasudotox	Not applicable, payload is a peptide molecule		

Belantamab mafodotin utilized novel MMAF payload which is primarily eliminated by fecal route in preclinical species. No organ impairment studies were reported for belantamab mafodotin, and this resulted in USFDA issuing post‐marketing requirement (PMR) for PK study in severe renal and moderate and severe hepatic impaired population. Similarly, trastuzumab emtansine utilized novel payload DM1 that is biliary‐fecal eliminated and did not report any organ impairment studies. USFDA issued a PMR to study PK of trastuzumab emtansine in patients with hepatic impairment. Sacituzumab govitecan also received a hepatic impairment related PMR since payload was novel, and organ impairment studies were not reported. Loncastuximab tesirine also received a similar PMR for hepatic impairment study.

Gemtuzumab ozogamicin was the first ADC to bring calicheamicin payload to the market, but did not report any organ impairment studies. Later, inotuzumab ozogamicin used the same linker‐payload and reported dedicated hepatic and renal impairment studies. It was reported that mild and moderate renal impairment and mild to moderate hepatic impairment did not significantly alter payload PK. The trials noted that there were not enough patients with severe hepatic impairment and only one with severe renal impairment. For renal impairment, inotuzumab ozogamicin did not recommend any dose modifications. For the hepatic impaired population, caution was recommended since the moderate and severe impairment data was not sufficient.

Trastuzumab deruxtecan did not report organ impairment studies in the BLA submission. However, the FDA did not issue any PMR or dose modifications in hepatic impaired populations. In the response, the FDA cited that the hepatic CYP3A4 and transporter DDI study yielded no significant change to payload PK, and this was used in a totality of evidence to conclude that moderate hepatic impaired patients can be administered trastuzumab deruxtecan safely without dose reduction.

## 4 Discussion

In recent years there have been multiple ADCs which have been ushered into the market as shown in Table 1. Since the safety and efficacy of the ADCs are driven primarily by payload exposure, it becomes immensely important to characterize ADME and DDI of the payload [56]. This involves generating in vitro and in vivo data, in silico modeling and occasionally clinical studies.

In vitro ADME characterization of the payloads is relatively straightforward and well‐established. Some of the physicochemical data regarding the payload, such as lipophilicity, protein binding and metabolic stability are established in the early discovery during compound screening. More specific ADME assays, including metabolite identification, CYP enzyme substrate, transporter substrate assessment are conducted prior to commencing clinical trials. Having information about payload metabolism and involvement of enzymes/transporters often is helpful in designing the clinical protocol for the first in human dosing. Payload being an inhibitor or inducer and acting as a precipitant of drug interactions is an unlikely scenario given the low circulating concentrations after ADC dosing. However, payload being a substrate of a CYP enzyme or drug transporter can certainly guide exclusion criteria for avoidance of certain concomitant medications during the ADC trial. Given the potency of the cytotoxic payloads, ADC therapeutics often have narrow therapeutic windows. Small changes in payload exposure can precipitate toxicities in patients. Similarly, drug interactions can also reduce payload exposure in early clinical trials and could potentially dilute the efficacy endpoints. This means early clinical investigations in smaller patient populations need to consider drug interactions and properly design the clinical protocols guided by the payload ADME data.

Preclinical in vivo ADME studies can be performed later when clinical studies are well underway. The in vivo studies are often performed in rodent and/or a nonrodent species and aim to quantify metabolism and excretion of the payload. The choice of rodent and nonrodent species is often dictated by toxicology studies and the in vitro metabolism data alignment between species. Based on published documents, monkeys are the large animal species of choice for preclinical mass balance studies for ADCs. A possible reason for the choice of monkeys could be their similarity to human physiology and cross‐reactivity of antibodies. Li et al. performed multiple species derived allometric scaling of human PK for ADCs and concluded that monkeys were the most suitable species for accurate predictions [57]. As mentioned in the FDA guidance “clinical pharmacology considerations for human mass balance studies,” ADC modalities can be exempt from suggestion of conducting clinical mass balance study. Guidance mentioned the risk of distributing radioactivity to critical organs in patients and suggested drawing conclusions based on preclinical studies. It was also mentioned to utilize in vitro ADME data and clinical nonradioactive sample analysis like nuclear magnetic resonance (NMR) spectroscopy or mass spectrometry in absence of clinical mass balance data [27].

Brentuximab vedotin is the only ADC that reported a clinical excretion study [58]. Polatuzumab vedotin and tisotumab vedotin did not report any additional in vivo mass balance or metabolism studies and used brentuximab vedotin as a reference. Enfortumab vedotin, however, reported a rat mass balance and excretion study with findings similar to brentuximab vedotin. As payload is expected to metabolize and eliminate from the body in a similar manner to previously approved ADC, rat mass balance study for enfortumab vedotin seemed redundant.

In contrast to mass balance, distribution studies for vedotin ADCs subsequent to brentuximab vedotin were performed independently. Tisotumab vedotin reported a PET imaging‐based distribution study in healthy monkeys as well as xenografted mice. Enfortumab vedotin reported ^3^H‐MMAE QWBA study in rats. Polatuzumab vedotin and telisotuzumab vedotin did not report any independent distribution studies. This repetition of distribution studies for the same linker‐payload stems from the fact that ADC distribution is a more complex phenomenon, and it is driven by target antigen, linker chemistry and payload physicochemical properties [59].

Distribution studies for ADCs are not clearly suggested in any of the regulatory guidance, and this has resulted in sponsors using best approaches on a case‐by‐case basis. Some studies focused on payload distribution, whereas others reported both antibody and payload distribution independently. ICH S9 guidance suggested that biodistribution studies for ADCs may not be warranted; however, sponsors reported some form of distribution data for several of the approved ADCs.

Another point of consideration in mass balance studies is in the position of the radio label on the molecule. Guidance clearly stated using label on the metabolically stable part of the molecule, and if needed, label two different positions on the molecule [27]. In preclinical ADC mass balance studies, sponsors have used a variety of approaches to handle this issue. For vedotin ADCs, the payload was radiolabeled, and the antibody and linker were not labeled. In the case of trastuzumab deruxtecan, both the payload and antibody were tagged with a different radioisotope, and experimental designs allowed studying the distribution of the antibody and payload independent of each other. Distribution of Sacituzumab govitecan in xenograft mice was studied using labeled antibody conjugated to cold payload. Mirvetuximab sorvatansine ADME studies used a radiolabel on the payload, and not on the antibody portion. For belantamab mafodotin, the biodistribution study was conducted using a fluorescent tagged antibody conjugated with cold payload. Studying the distribution of an ADC without labeling a payload is a less helpful exercise when characterizing a cytotoxic payload. These studies may yield data on target expression and tumor indication selection, but do not provide good data for payload distribution. If the linker used in the ADC is noncleavable, then the linker can also be considered for the position of the radiolabel. Of the 15 ADCs presented in this review, none have performed preclinical mass balance studies with a tracer label on the linker portion of the molecule. Guidelines will need to suggest specific design considerations for biodistribution studies for ADCs to avoid these issues. Additionally, the regulator's perspective on nonradiolabel tracers will be helpful in terms of cost and safety associated with biodistribution studies.

Concentrations of the payload in circulation are generally smaller given the design of the ADC, and that justifies USFDA's overall approach about payload as a precipitant of drug interactions. Although sponsors are expected to perform in vitro enzyme and transporter inhibition and enzyme induction experiments, it's understood that these effects are not plausible at clinically relevant concentrations of the payloads.

On the contrary, payload as a substrate of drug interactions has been evaluated in depth. Sponsors have used two distinct approaches to address payload substrate drug interaction risk assessment. The first approach is to design and conduct a clinical DDI study in patients. Brentuximab vedotin and trastuzumab deruxtecan reported dedicated DDI studies and designed the language on the prescribing information based on the results of the DDI study. Trastuzumab emtansine and mirvetuximab sorvatansine did not report clinical DDI studies and relied on the in vitro ADME data for the prescribing label. The prescribing information suggested close monitoring of patients when on concomitant CYP3A4 inhibitor medications with trastuzumab emtansine [11].

This second approach of not performing clinical DDI study and providing exclusions on the label is also utilized by Sacituzumab govitecan. The payload SN38 is metabolized by UGT1A1, and the sponsor collected UGT genotyping information prospectively. However, the DDI‐related warning was added to the label based on in vitro data. Assessment of limited patients from Phase 1 is outlined in EMEA product information, for no change in exposure of SN38 due to UGT1A1 inhibitors and inducers [60]. Exclusion for strong CYP3A inhibitors and inducers was also indicated in the absence of clinical DDI studies.

Trastuzumab deruxtecan conducted clinical study for CYP3A and OATP1B1 inhibition. Although the payload Dxd is metabolically stable and is excreted ~99% unchanged in preclinical studies, the DDI study with CYP3A inhibitor was conducted. According to in vitro studies, the payload of trastuzumab emtansine is highly metabolized, and CYP3A4/5 enzymes are involved in its metabolism, but the clinical DDI study with CYP3A inhibitor was not reported.

Loncastuximab tesirine reported that payload SG3199 is a substrate for CYP3A enzymes as well as Pgp transporter. In vivo metabolism studies were not reported, and the extent of metabolism in vitro or in vivo was not specified for the payload. Interestingly, despite all the in vitro data reported, the prescribing information does not have any warnings or precautions mentioned about concomitant administration of CYP3A or Pgp inhibitors. The label does mention precautions in the population with hepatic impairment, possibly because of hepatic metabolism and elimination of SG3199, but specifically DDI related warning is not provided. PopPK analysis of patients on concomitant Pgp inhibitors was reported, and it was noted that total antibody and conjugated antibody PK remained unaffected [16]. This was possibly a retrospective covariate analysis and not a dedicated clinical DDI study. Gemtuzumab ozogamicin also did not perform any clinical DDI studies despite the payload being extensively metabolized. In the reported information to the regulators, sponsors provided the preclinical in vivo data depicting extensive metabolism and hepatobiliary excretion of the payload. FDA requested post‐marketing studies to further investigate enzymes responsible for payload metabolism, and any subsequent DDI evaluations. These diverse approaches taken by sponsors to essentially answer the same DDI question, primarily about CYP3A enzyme, can be more aligned in future with more direct guidelines from regulators.

For the linker‐payloads that are approved and then reused in another ADC, the DDI assessment is quite straightforward. For datopotamab deruxtecan, the sponsor has described the PBPK modeling efforts for this payload based on DDI data from trastuzumab deruxtecan. For any given payload, once the clinical DDI data are available, the validation of the PBPK model is performed based on that data, and independent DDI investigations are not required. If the linker‐payload and DAR of the new ADC is similar to previously approved ADC, then even the doses are in the similar range, and PBPK assessment may also be unnecessary. Tisotumab vedotin used this approach, since the linker‐payload and DAR were exactly the same, and clinical doses were also in a similar range to brentuximab vedotin.

USFDA approach in terms of organ impairment studies for ADCs is more consistent compared to DDI and other ADME data requirements. Almost all small molecule payloads from approved ADCs are eliminated primarily through the biliary‐fecal route. Some sponsors reported dedicated clinical hepatic impairment studies, and their product labels were guided by the relevant data. For those sponsors who did not provide the organ impairment data, PMRs were issued specifically based on provided in vitro and preclinical in vivo data.

For hepatic impairment studies for ADCs, USFDA points the sponsors to use 2003 guidance [39], as well as organ impairment in patients with malignancy [40]. 2003 guidance recommends sponsors to use Child‐Pugh score as a measure of hepatic function; however, in the PMR that have been issued for mirvetuximab sorvatansine and other ADCs, a different metric from NCI‐ODWG was suggested. Child‐Pugh score is a more detailed and resource‐intensive measurement of liver function compared to NCI ODWG, and this could affect patient enrollment in the trials. With evolution in USFDA's thinking on the liver function metric in oncology patients, the hepatic impairment study guidance also needs an update. In September 2026, USFDA released a draft guidance for PK studies in hepatic impaired patients, and this addressed the Child‐Pugh vs. NCI ODWG measurement for liver function. This guidance is not cited here since it's still in the draft form.

For trastuzumab deruxtecan, the hepatic impairment study was not reported even though the payload is primarily eliminated by the biliary‐fecal route. Additionally, the label did not state any dose modification for patients with moderate hepatic impairment [13]. USFDA considered lack of clinical DDI with CYP3A4, P‐gp and OATP inhibitors with trastuzumab deruxtecan as a supporting information to waive hepatic impairment dose modifications.

## 5 Future Directions

As a preliminary effort to understand the scope and interpretation of the FDA guidance, a literature search for the data of 15 approved ADCs was performed. For this purpose, data available in published literature including regulatory submissions, prescribing information, and research papers were reviewed. These case studies shed light on the methods each sponsor selected to address the DDI‐related questions based on existing guidance. It also brings forth the complexity and challenges specifically faced by sponsors when introducing novel payloads for ADCs.

In Figure 2, we have elaborated a general approach for payload ADME characterization at different stages of ADC development. For the new cytotoxic payloads, the in vitro assays can be performed in discovery and early development stages. The preclinical in vivo studies including mass balance can be performed for a payload in rats, and for ADC in monkeys, and use of radioactivity tracer is recommended on payload and not on antibody portion of the molecule. Similarly, distribution studies should be performed in monkeys if the target is expressed. If monkeys do not express the target, then another species can be considered with cross‐reactivity. However, given that ADCs are known to distribute to nontarget‐expressing tissues as well [61] distribution study in monkeys can still be beneficial to understand off‐target toxicities. Kraynov et al. [25] agree with this approach in the white paper discussing the ADME studies for ADCs. These intricacies in study design need to be guided by more detailed recommendations from the regulators. For biodistribution studies with ADC administration, the tracer label on the metabolically stable portion of the payload molecule is suggested.

**FIGURE 2:**
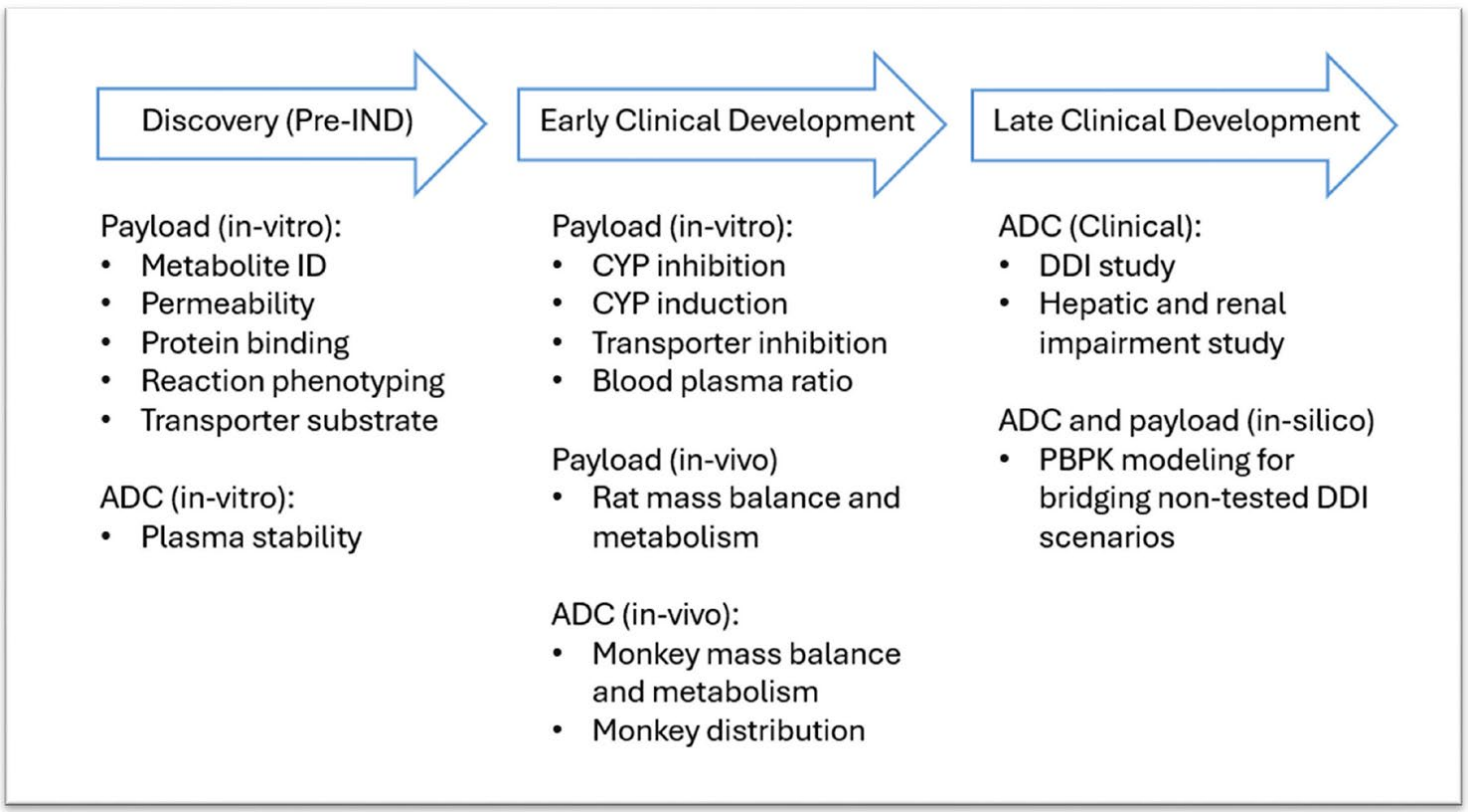
Typical ADME studies during development of antibody‐drug conjugate.

The overall landscape is evolving fast for ADCs and will continue to change as newer ADCs get introduced on the market. Based on our literature review, we can clearly see that the approach to establish ADME for the payloads has been unique and varied. Additionally, the FDA guidance provides room for interpretation, thus broadening the scope but providing limited specifics. Moreover, emerging novel payloads and the next generation of ADCs pose newer challenges for ADME scientists. Choice of animal species for in vivo ADME studies, different approaches for biodistribution studies, multiple options for the radiolabel of ADC, and so on. Varying paths taken by different sponsors to reach the same goal provide an uncertain path for a novel payload developer. However, in due course of time, this guidance will evolve to meet the changing needs of the industrial landscape and clarify regulator suggestions for ADCs to make those as clear as they are for the small molecule modalities.

## Author Contributions

**Vijay More:** conceptualization, writing – original draft, writing – review and editing, visualization. **Kiran Deshpande:** writing – original draft, writing – review and editing, visualization. **Chaitali Passey:** writing – review and editing, conceptualization. **Gaurav Bajaj:** writing – review and editing, conceptualization. **Jeff Barrett:** writing – review and editing, conceptualization. **Manish Gupta:** writing – review and editing, conceptualization.

## Conflicts of Interest

The authors declare no conflicts of interest.

## Data Availability

Data sharing not applicable to this article as no datasets were generated or analysed during the current study.

## References

[1] M. Walles , A. Connor , and D. Hainzl , “ADME and Safety Aspects of Non‐Cleavable Linkers in Drug Discovery and Development,” Current Topics in Medicinal Chemistry 17, no. 32 (2017): 3463–3475, 10.2174/1568026618666180118153502. 29357804

[2] R. Wang , B. Hu , Z. Pan , et al., “Antibody–Drug Conjugates (ADCs): Current and Future Biopharmaceuticals,” Journal of Hematology & Oncology 18, no. 1 (2025): 51, 10.1186/s13045-025-01704-3. PMC12044742 40307936

[3] Pfizer , “Adcetris Pfizer Prescribing Information 2025,” accessed September 2, 2025, https://www.pfizermedical.com/adcetris/clinical‐pharmacology?utm_source=chatgpt.com#ID_4504f5f4‐b0b9‐5533‐6eb5‐44f23509a5c6.

[4] FDA , “Polatuzumab Vedotin (BLA 761121)” (2018).

[5] FDA , “Enfortumab Vedotin (BLA 761137)” (2019).

[6] Astellas , “Enfortumab Vedotin Label” (2019).

[7] FDA , “Tisotumab Vedotin (BLA 761208)” (2021).

[8] FDA , “Telisotuzumab Vedotin‐Tllv (BLA 761384)” (2024).

[9] GlaxoSmithKline , “Belantamab Mafodotin Product Monograph” (2025).

[10] EMA , “Trastuzumab Emtansine Product Information” (2013).

[11] FDA , “Mirvetuximab Soravtansine (BLA 761310)” (2022).

[12] EMA , “Mirvetuximab Soravtansine Product Information” (2024).

[13] FDA , “Trastuzumab Deruxetecan (BLA 761139)” (2019).

[14] FDA , “Daptopotomab Deruxetecan (BLA 761394)” (2024).

[15] FDA , “Sacituzumab Govitecan (BLA 761115)” (2019).

[16] FDA , “Loncastuximab Tesirine‐lpyl (BLA 761196)” (2020).

[17] FDA , “Moxetumomab Pasudotox (BLA 761104)” (2017).

[18] FDA , “Inotuzumab Ozogamicin (BLA 761040)” (2016).

[19] Pfizer , “Gemtuzumab Ozogamicin Product Monograph” (2019).

[20] Pfizer , “Gemtuzumab Ozogamicin Label” (2023).

[21] D. Su and D. Zhang , “Linker Design Impacts Antibody‐Drug Conjugate Pharmacokinetics and Efficacy via Modulating the Stability and Payload Release Efficiency,” Frontiers in Pharmacology 12 (2021): 687926, 10.3389/fphar.2021.687926. PMC8262647 34248637

[22] FDA , “Clinical Pharmacology Considerations for Antibody‐Drug Conjugates Guidance for Industry” (2024), accessed November 30, 2025, https://www.fda.gov/regulatory‐information/search‐fda‐guidance‐documents/clinical‐pharmacology‐considerations‐antibody‐drug‐conjugates‐guidance‐industry.

[23] JPMA , Technical Guidelines for Non‐Clinical Studies of Antibody‐Drug Conjugates (JPMA, 2023).

[24] NMPA , Technical Guideline for Nonclinical Studies of Antibody–Drug Conjugates (NMPA, 2023).

[25] E. Kraynov , A. V. Kamath , M. Walles , et al., “Current Approaches for Absorption, Distribution, Metabolism, and Excretion Characterization of Antibody‐Drug Conjugates: An Industry White Paper,” Drug Metabolism and Disposition 44, no. 5 (2016): 617–623, 10.1124/dmd.115.068049. 26669328

[26] NRCILAR , Science, Medicine, and Animals (National Academies Press, 2004). 20669472

[27] FDA , “Clinical Pharmacology Considerations for Human Radiolabeled Mass Balance Studies” (2024), accessed November 30, 2025, https://www.fda.gov/regulatory‐information/search‐fda‐guidance‐documents/clinical‐pharmacology‐considerations‐human‐radiolabeled‐mass‐balance‐studies.

[28] B. Rudolph , J. A. Davis , D. Hainzl , and M. Walles , “A General Perspective for the Conduct of Radiolabelled Distribution, Metabolism, and Excretion Studies for Antibody‐Drug Conjugates,” Xenobiotica 54, no. 8 (2024): 521–532, 10.1080/00498254.2024.2336576. 39329287

[29] N. Jain , S. W. Smith , S. Ghone , and B. Tomczuk , “Current ADC Linker Chemistry,” Pharmaceutical Research 32, no. 11 (2015): 3526–3540, 10.1007/s11095-015-1657-7. PMC4596905 25759187

[30] R. Mathur and G. J. Weiner , “Picking the Optimal Target for Antibody‐Drug Conjugates,” American Society of Clinical Oncology Educational Book. American Society of Clinical Oncology. Annual Meeting (2013). 10.14694/EdBook_AM.2013.33.e103 23714470

[31] J. K. Simmons , P. J. Burke , J. H. Cochran , P. G. Pittman , and R. P. Lyon , “Reducing the Antigen‐Independent Toxicity of Antibody‐Drug Conjugates by Minimizing Their Non‐Specific Clearance Through PEGylation,” Toxicology and Applied Pharmacology 392 (2020): 114932, 10.1016/j.taap.2020.114932. 32109510

[32] E. Khera , S. Dong , H. Huang , L. de Bever , F. L. van Delft , and G. M. Thurber , “Cellular‐Resolution Imaging of Bystander Payload Tissue Penetration From Antibody‐Drug Conjugates,” Molecular Cancer Therapeutics 21, no. 2 (2022): 310–321, 10.1158/1535-7163.MCT-21-0580. PMC8832507 34911819

[33] FDA , “S9 Nonclinical Evaluation for Anticancer Pharmaceuticals” (2010).

[34] FDA , “M12 Drug Interaction Studies” (2024), accessed November 30, 2025, https://www.fda.gov/regulatory‐information/search‐fda‐guidance‐documents/m12‐drug‐interaction‐studies.

[35] FDA , “Drug–Drug Interaction Assessment for Therapeutic Proteins Guidance for Industry” (2023), accessed November 30, 2025, https://www.fda.gov/regulatory‐information/search‐fda‐guidance‐documents/drug‐drug‐interaction‐assessment‐therapeutic‐proteins‐guidance‐industry.

[36] C. Li , R. Menon , M. Walles , et al., “Risk‐Based Pharmacokinetic and Drug–Drug Interaction Characterization of Antibody–Drug Conjugates in Oncology Clinical Development: An International Consortium for Innovation and Quality in Pharmaceutical Development Perspective,” Clinical Pharmacology & Therapeutics 112, no. 4 (2022): 754–769, 10.1002/cpt.2448. 34657311

[37] D. Samineni , H. Ding , F. Ma , et al., “Physiologically Based Pharmacokinetic Model‐Informed Drug Development for Polatuzumab Vedotin: Label for Drug‐Drug Interactions Without Dedicated Clinical Trials,” Journal of Clinical Pharmacology 60 (2020): S120–S131. 10.1002/jcph.1718 33205435

[38] M. P. Choules , P. Zuo , Y. Otsuka , A. Garg , M. Tang , and P. Bonate , “Physiologically Based Pharmacokinetic Model to Predict Drug–Drug Interactions With the Antibody–Drug Conjugate Enfortumab Vedotin,” Journal of Pharmacokinetics and Pharmacodynamics 51, no. 5 (2024): 417–428, 10.1007/s10928-023-09877-5. PMC11576838 37632598

[39] FDA , “Pharmacokinetics in Patients With Impaired Hepatic Function: Study Design, Data Analysis, and Impact on Dosing and Labeling” (2003).

[40] FDA , “Cancer Clinical Trial Eligibility Criteria: Patients With Organ Dysfunction or Prior or Concurrent Malignancies” (2020).

[41] J. A. Davis , D. A. Rock , L. C. Wienkers , and J. T. Pearson , “In Vitro Characterization of the Drug‐Drug Interaction Potential of Catabolites of Antibody‐Maytansinoid Conjugates,” Drug Metabolism and Disposition 40, no. 10 (2012): 1927–1934, 10.1124/dmd.112.046169. 22752008

[42] H. Okamoto , M. Oitate , K. Hagihara , et al., “Pharmacokinetics of Trastuzumab Deruxtecan (T‐DXd), a Novel Anti‐HER2 Antibody‐Drug Conjugate, in HER2‐Positive Tumour‐Bearing Mice,” Xenobiotica 50, no. 10 (2020): 1242–1250, 10.1080/00498254.2020.1755909. 32306807

[43] N. Hanioka , S. Ozawa , H. Jinno , et al., “Interaction of Irinotecan (CPT‐11) and Its Active Metabolite 7‐Ethyl‐10‐Hydroxycamptothecin (SN‐38) With Human Cytochrome P450 Enzymes,” Drug Metabolism and Disposition 30, no. 4 (2002): 391–396. 10.1124/dmd.30.4.391 11901092

[44] X.‐Y. Chu , H. Suzuki , K. Ueda , Y. Kato , S.‐I. Akiyama , and Y. Sugiyama , “Active Efflux of CPT‐11 and Its Metabolites in Human KB‐Derived Cell Lines,” Journal of Pharmacology and Experimental Therapeutics 288, no. 2 (1999): 735–741, 10.1016/S0022-3565(24)38014-0. 9918583

[45] V. Yip , O. M. Saad , D. Leipold , C. Li , A. Kamath , and B. Q. Shen , “Monomethyl Auristatin E (MMAE), a Payload for Multiple Antibody Drug Conjugates (ADCs), Demonstrates Differential Red Blood Cell Partitioning Across Human and Animal Species,” Xenobiotica 54, no. 8 (2024): 511–520, 10.1080/00498254.2024.2345849. 38647387

[46] Pfizer , “Brentuximab Vedotin Prescribing Information” (2011), accessed October 9, 2025, https://www.adcetris.com/classical‐hodgkin‐lymphoma/?cmp=163267313431&utm_source=GOOGLE&utm_medium=paidsearch&pid=Brand&utm_term=brentuximab%20vedotin&network=g&gad_source=1&gad_campaignid=21140366045&gbraid=0AAAAADi6WJCR0EeoIT7NtJLbanPp7I9mc&gclid=EAIaIQobChMIt4CR3caYkAMV7UpHAR2‐Zy92EAAYASAAEgL1NfD_BwE&gclsrc=aw.ds.

[47] Pfizer , “Brentuximab Vedotin Label” (2011).

[48] S. Takahashi , M. Karayama , M. Takahashi , et al., “Pharmacokinetics, Safety, and Efficacy of Trastuzumab Deruxtecan With Concomitant Ritonavir or Itraconazole in Patients With HER2‐Expressing Advanced Solid Tumors,” Clinical Cancer Research 27, no. 21 (2021): 5771–5780, 10.1158/1078-0432.CCR-21-1560. PMC9401457 34426442

[49] Daiichi‐Sankyo , “Trastuzumab Deruxtecan Label” (2019).

[50] T. Nakamura , M. Shimada , M. Takabayashi , et al., “Drug‐Drug Interaction Between Trastuzumab Emtansine (T‐DM1) and Orally Administered Tacrolimus in a Patient and in Rats,” Drug Metabolism and Pharmacokinetics 62 (2025): 101486, 10.1016/j.dmpk.2025.101486. 40513507

[51] J. B. Lee , S. Zhou , M. Chiang , X. Zang , T. H. Kim , and L. Kagan , “Interspecies Prediction of Pharmacokinetics and Tissue Distribution of Doxorubicin by Physiologically‐Based Pharmacokinetic Modeling,” Biopharmaceutics & Drug Disposition 41, no. 4–5 (2020): 192–205, 10.1002/bdd.2229. 32342986

[52] S.‐j. Rhee , H. Chung , S. Yi , K.‐S. Yu , and J.‐Y. Chung , “Physiologically Based Pharmacokinetic Modelling and Prediction of Metformin Pharmacokinetics in Renal/Hepatic‐Impaired Young Adults and Elderly Populations,” European Journal of Drug Metabolism and Pharmacokinetics 42, no. 6 (2017): 973–980, 10.1007/s13318-017-0418-x. 28536774

[53] Y. Chen , D. Samineni , S. Mukadam , et al., “Physiologically Based Pharmacokinetic Modeling as a Tool to Predict Drug Interactions for Antibody‐Drug Conjugates,” Clinical Pharmacokinetics 54, no. 1 (2015): 81–93, 10.1007/s40262-014-0182-x. 25223698

[54] FDA , “Belantamab Mafodotin‐Blmf (BLA 761158),” FDA Website (2020).

[55] B. Zhao , R. Chen , O. A. O'Connor , et al., “Brentuximab Vedotin, an Antibody–Drug Conjugate, in Patients With CD30‐Positive Haematologic Malignancies and Hepatic or Renal Impairment,” British Journal of Clinical Pharmacology 82, no. 3 (2016): 696–705, 10.1111/bcp.12988. PMC5089583 27115790

[56] R. S. Foti , R. F. Tyndale , K. L. Garcia , et al., ““Target‐Site” Drug Metabolism and Transport,” Drug Metabolism and Disposition 43, no. 8 (2015): 1156–1168, 10.1124/dmd.115.064576. PMC11024933 25986849

[57] C. Li , C. Zhang , R. Deng , et al., “Prediction of Human Pharmacokinetics of Antibody–Drug Conjugates From Nonclinical Data,” Clinical and Translational Science 12, no. 5 (2019): 534–544, 10.1111/cts.12649. PMC6742937 31115997

[58] FDA , “Brentuximab Vedotin (BLA 125388)” (2022).

[59] P. J. Burke , J. Z. Hamilton , S. C. Jeffrey , et al., “Optimization of a PEGylated Glucuronide‐Monomethylauristatin E Linker for Antibody–Drug Conjugates,” Molecular Cancer Therapeutics 16, no. 1 (2017): 116–123, 10.1158/1535-7163.MCT-16-0343. 28062707

[60] EMA , “Sacituzumab Govitecan Product Information” (2021).

[61] B. Wilkin , “Off‐Target Toxicity in Antibody‐Drug Conjugates ‐ Crown Bioscience” (2025).

